# Monitoring Acid–Base Titrations on Wax Printed Paper Microzones Using a Smartphone

**DOI:** 10.3390/mi8050139

**Published:** 2017-05-02

**Authors:** Sandro A. Nogueira, Lucas R. Sousa, Nathália K. L. Silva, Pedro H. F. Rodrigues, Wendell K. T. Coltro

**Affiliations:** 1 Instituto de Química, Universidade Federal de Goiás, Campus Samambaia, Goiânia, GO 74690-900, Brazil; sandro@ufg.br (S.A.N.); l.rod.sousa@gmail.com (L.R.S.); nathalia.karoline.lsilva@gmail.com (N.K.L.S.); phfr94@hotmail.com (P.H.F.R.); 2 Instituto Nacional de Ciência e Tecnologia em Bioanalítica, Campinas, SP 13084-971, Brazil

**Keywords:** cell-phone analysis, chemical education, instrument-free chemical assays, microfluidic paper-based analytical devices, paper microfluidics, spot tests

## Abstract

This study describes the use of a smartphone for monitoring acid–base titrations on wax printed paper microzones. An array of twelve microzones of 5 mm diameter each was wax printed on filter paper. The analytical performance of the proposed devices was explored with acid–base titrations examples, where jaboticaba peel extract was used as a natural pH indicator. The color intensity was captured using a smartphone and analyzed through a free App named Photometrix^®^. Before titrations, color intensity versus pH was calibrated to be used as a reference in titrations as (i) strong acid versus strong base; (ii) strong base versus strong acid; and (iii) weak acid versus strong base. In all examples, images were obtained after the addition of each aliquot of titrant solutions. The obtained titration curves showed the same behavior as the conventional titration curves. After evaluating the feasibility of the proposed methodology, the concentration level of acetic acid was obtained in three vinegar samples. Although the obtained values ranged from 5% to 8% compared to the concentrations on the conventional method, the proposed methodology presented high analytical reliability. The calculated concentrations of acetic acid in three samples ranged from 3.87% to 3.93%, and the proposed methodology did not significantly differ from classic acid–base titration at a confidence level of 95%. The acid–base titration on paper-based devices is outstanding, since any titration can be completed within 5 min using 20 µL volumes. Besides, the use of a smartphone to capture images followed by analysis in a free app offers simplicity to all users. The proposed methodology arises as a new strand to be exploited in the diffusion of the analytical chemistry education field as well as an alternative for quantitative analysis with extremely simplified instrumentation.

## 1. Introduction

Microfluidic paper-based devices (µPADs) have received considerable attention from the scientific community. This platform offers great advantages, including biocompatibility, low cost, global affordability, and especially the capability to perform tests under lateral or vertical flow with reduced consumption of reagents and samples [1,2,3,4]. Since the first publications from Whitesides’ group [5,6], µPADs have been explored for clinical [1,3], biological [7], food [8], environmental [9], chemical sensing [10], and forensic [11] applications. Recent examples of applications using µPADs have demonstrated the ease in coupling with different detectors, including mass spectrometry [12,13], chemiluminescence [14], fluorescence [15], electrochemical methods [16,17,18], and colorimetric detection [6,19,20]. The latter offers instrumental simplicity and portability, once digital images can be recorded through popular electronic devices like smartphones, digital cameras, or scanners [6,21].

The use of smartphones for analytical applications in conventional and miniaturized scales has significantly increased in recent years [6,20,22,23]. As recently observed in the literature, the combination of smartphones and microfluidic devices has promoted a real explosion in the number of publications, and it is a global trend—especially in laboratories with limited resources or restricted access to sophisticated infrastructure. However, the main challenges associated with colorimetric detection by smartphone are external light control and the technical ability of the user to capture images, which can both compromise the reliability of the analytical response [20,21]. Some apps—which are not very widespread in publications associated with microfluidics—are dedicated to colorimetric detection (e.g., Colormeter^®^ and Photometrix^®^). The latter was recently developed by Helfer and co-workers [24], and it is compatible with Android and Windows platforms. Basically, the app’s goals are to capture information about color intensity and provide chemometric analysis. Photometrix^®^ is an application for the univariate calibration and exploratory analysis of multivariate data from the decomposition of acquired digital images. The decomposition generates the pixel’s intensity in eight channels extracted from different color models [24]. This application presents a user-friendly interface and it can be obtained for free in the Google Play Store. 

Recently, some research groups have explored the paper platform to perform acid–base and redox titrations based on colorimetric measurements. Karita and Kaneta reported acid–base titrations on µPADs using an array with ten microzones for reaction and detection interconnected to a central zone for sample inlet [25]. While reaction zones were preloaded with different concentrations of primary standard solutions, detection zones were spotted with colorimetric indicator. After the addition of 30 μL aliquots of solution at the sample inlet zone, the fluid was transported through the channel by lateral flow reaching the reaction and detection zones. The endpoint of titration was determined by naked eye based on color changes which are associated with the detection of excess amounts of base or acid. Two limitations of the method reported by Karita and Kaneta are related to the poor color uniformity and stability after drying, as well as the dependence of the indicator concentration for a better visualization of the endpoint of titration [25]. In 2015, Myers and co-workers reported an iodine titration on µPAD [26]. Color intensity was captured through a scanner and analyzed in graphics software. 

In this report, we describe the use of a free smartphone application to monitor acid–base titrations on wax printed paper microzones. Acid–base reactions were monitored using a natural pH indicator prepared from jaboticaba peel extract, which provides a color gradient over a wide pH range. The color intensity for each pH was calibrated and used to monitor titrations involving (i) strong acid versus strong base; (ii) strong base versus strong acid; and (iii) weak acid versus strong base. The feasibility of the proposed approach for performing quantitative analysis of acetic acid concentrations in vinegar samples was successfully demonstrated. Differently from the mentioned references [25,26], the current study makes use of a smartphone equipped with a free App to monitor acid–base titrations on wax printed paper zones. The proposed method was able to monitor not only the endpoint of the titration, but also the color intensity over the wide pH range. The instrumental simplicity and requirements enable their use for experimental practices in high school or undergraduate courses. Regarding the selection of sample, we chose vinegar because the determination of the acetic acid concentration in this sample is a common practice to teach principles of quantitative analysis for students of different levels or courses.

## 2. Materials and Methods 

### 2.1. Materials

Hydrochloric acid (Synth, Diadema, São Paulo, Brazil), sodium hydroxide (Vetec, Duque de Caxias, Rio de Janeiro, Brazil), potassium hydrogen phthalate (Synth, Diadema, São Paulo, Brazil), ethanol (Vetec, Duque de Caxias, Rio de Janeiro, Brazil), sodium tetraborate (Synth, Diadema, São Paulo, Brazil), phenolphthalein (Synth, Diadema, São Paulo, Brazil), and methyl orange (Isofar, Duque de Caxias, Rio de Janeiro, Brazil) were used without any purification. Filter paper model JP40 was obtained from Quanty (São José dos Pinhais, Paraná, Brazil).

### 2.2. Fabrication of Wax Printed Microzones

**Figure 1:**
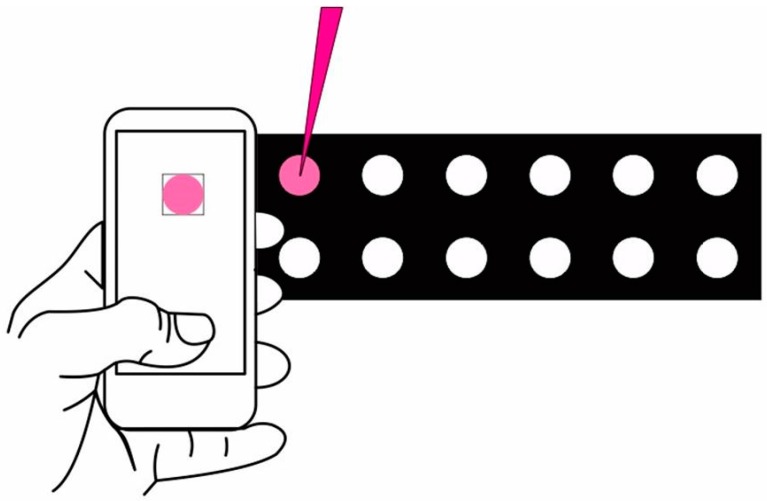
Scheme of paper devices for acid–base reactions and procedure for colorimetric reading by smartphone.

Paper microzones were prepared in quantitative filter paper JP40 by wax printing [27]. In this study, twelve microzones with 5 mm diameter each, distributed in two rows of six microzones each, were drawn on Corel Draw software and printed on paper by using a wax printer (Xerox ColorQube 8570, Xerox Corporation, Rochester, NY, USA). After printing, devices were passed three times in a laminator heated to 150 °C to completely melt the wax and thus create effective hydrophobic barriers on paper substrates. The lamination step was performed at rate of 60 cm/min. Adhesive tape was fixed to the device bottom to avoid solution leakage. Figure 1 displays the fabricated device layout.

### 2.3. Calibration of the Color Intensity and Acid–Base Titrations

Acid–base titrations were performed on wax printed microzones using jaboticaba peel extract (*Myrciaria califlora*) as a natural pH indicator. For this purpose, 5 µL of the natural indicator was added to the zones and allowed to dry at room temperature for 5 min. Prior to titrations, the calibration of the colorimetric response over the pH range was performed by adding 5 µL aliquots of different standard solutions into the microzones spotted with the indicator. Standard solutions were prepared in a pH range between 1 and 12. Table S1 (available in the supplementary material) presents the composition of all analyzed solutions. For titration, microzones were also spotted with 5 µL of the natural indicator, as previously mentioned. Then, 1.2 µL aliquots of the titrant solution (0.1 mol/L acid or base solution) were successively added into the microzones, and a digital image was recorded between each addition. 

### 2.4. Indicator Preparation

The natural indicator was prepared according to the procedure described by Guimarães and co-workers [28]. Briefly, five jaboticaba fruits were collected, corresponding to a mass of ca. 34.4 g. The fruits were immersed in 100 mL of 96% ethanol (Vetec, Duque de Caxias, Rio de Janeiro, Brasil) for 6 h. The obtained extract was filtered and kept in a dark flask to minimize light influence, which degrades anthocyanins. 

### 2.5. Image Capture and Colorimetric Analysis

Images were captured with a Samsung Galaxy smartphone model J5 equipped with a 13 MP resolution camera (Samsung Electronics, Suwon, Gyeonggi, Korea). Images were directly captured using the Photometrix^®^ App, available for free download on the Google Play Store. All images were captured with ambient light, keeping the smartphone at distance of 10 cm from the wax printed paper device. The region of interest (ROI) containing 64 pixels × 64 pixels was selected for each image. ROIs were decomposed in the following color channels: red (R), green (G), blue (B), hue (H), saturation (S), value (V), lightness (L), and intensity (I), providing the pixel’s average value [24].

### 2.6. Quantitative Analysis of Acetic Acid in Vinegar Samples

The proposed approach was investigated as a portable and disposable volumetric platform for quantitative analysis. As proof-of-concept, the acetic acid concentration level was determined in three different vinegar brands and compared to the values achieved by a standard titration method. Paper-based assays were carried out without any previous sample treatment; i.e., vinegar sample aliquots were added to the microzones and analyzed without dilution. 

## 3. Results and Discussion

The use of paper-based microzones prepared by wax printing for applications involving acid–base titrations opens a new gate to be explored in the chemistry field, including the possibility to perform experiments in analytical chemistry laboratories or even basic chemistry for high school. The advantages associated with paper-based devices as well as the popularity of smartphones make their application in any laboratory or research center with limited-resources possible.

Microzones were initially fabricated with diameter ranging between 1 and 8 mm. Through the wax printing method, zones with diameter between 2 and 8 mm can be produced. For 1-mm-diameter, wax particles promote partial or total blockage of the zones. Better resolution was observed for diameters higher than 2 mm. During the investigation, the use of zones with diameter between 2 and 4 mm compromises the quality of captured image due to the poor focus adjust. For colorimetric analysis, the best focus for image capture was achieved using zones defined with 5 mm diameter. Similar results were achieved for zones defined with larger diameters; however, they require a greater volume of sample or reagents. For this reason, we decided to use zones with 5 mm diameter and capture the image at a distance of 10 cm.

### 3.1. Natural Indicator Choice

When compared to a previous report from Karita and Kaneta [25], the use of a natural indicator composed of anthocyanins extracted from jaboticaba to monitor acid–base titrations is advantageous as it promotes a color gradient through a wide pH range, varying from magenta to different shades of green. As previously reported [25], the use of synthetic indicators like phenolphthalein, methyl orange, and bromocresol purple offer color changes in a narrow pH range, which is dependent of the pK of each indicator. In addition, natural indicators have toxicity lower than synthetic indicators, causing less environmental impact. The indicator lifetime in solution is ca. 5 months if stored either at room temperature or when kept refrigerated. However, it needs to be kept in a dark and closed flask to avoid contamination or ethanol evaporation, which would lead to indicator pre-concentration and, consequently, changes in the color intensity. When spotted on paper, the indicator lifetime is considerably reduced due to some factors that influence its stability, such as light and temperature [29,30]. To estimate the stability, microzones were spotted with the indicator and the color intensity was recorded between 10 min and 24 h. Based on the colorimetric analysis, the relative standard deviation (RSD) ranged from 1.3% to 3.0%. For experimental practices, this low RSD suggests that the microzones can be wax printed, spotted with colorimetric indicator, and then explored by different student groups or even classes of experimental chemistry over the course of 1 day, for example. The stability over longer times was not evaluated because the printed platform can be quickly prepared by students at the beginning of each experiment.

### 3.2. Calibration of the Colorimetric Response versus pH Range

**Figure 2:**
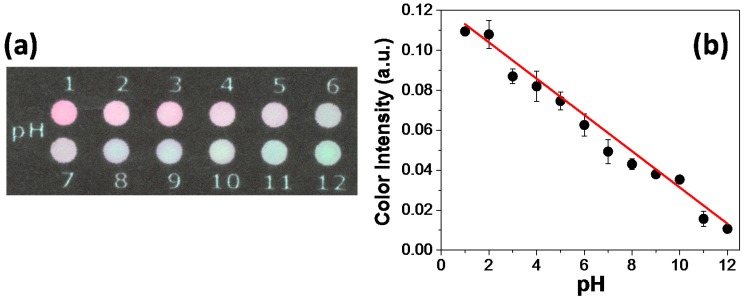
Presentation of (**a**) an optical micrograph showing the color changes on printed zones and (**b**) color intensity analysis over different pH values. In (a), microzones were first spotted with 5 µL of the natural indicator. Then, 5 µL of different solutions prepared at pH range from 1 to 12 were added on microzones prior to image capture. The labels depicted in (a) indicate the used microzones for each pH value.

Before proceeding acid–base titrations on paper zones, the colorimetric response to each pH value was evaluated to establish a color pattern as reference. As observed in the optical micrograph depicted in Figure 2a, the pH variation for values between 1 and 12 leads to a color change from magenta to green. The data associated with the color intensity versus pH is presented in Figure 2b. The analytical response was obtained by using color information from channel S. This channel measures the color saturation (i.e., the amount of color that is present in the ROI) [24]. The achieved data were linearly fitted and presented a coefficient of determination equal to 0.99. As it can be noted in Figure 2b, a better correlation for pH values between 2 and 11 can be inferred. For pH values lower than 2 and higher than 11, the color intensity did not exhibit appreciable changes. This may be attributed to the indicator stability in extreme pH values. 

**Figure 3:**
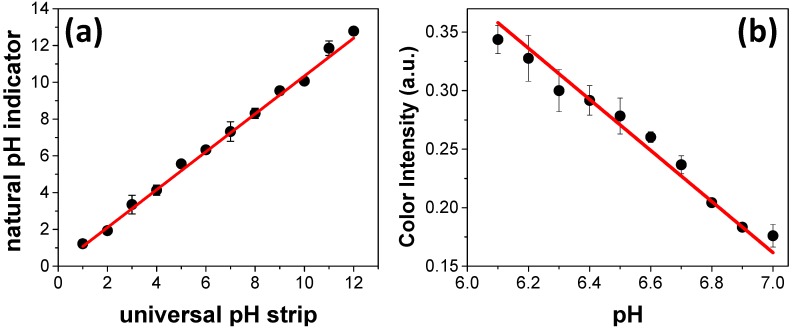
Presentation of (**a**) pH values measured with universal pH strip and wax printed paper zones previously spotted with natural pH indicator and (**b**) pH resolution measured on paper zones. In (a), all measurements were recorded for standard solutions prepared in a pH range between 1 and 12. For the readings using universal pH strips, each device was introduced inside sample solution requiring a volume of ca. 1 mL to obtain coloration and allow the comparison with a pH scale defined in a color gradient as reference. In (b), the color intensity was determined in phosphate buffer solutions prepared in a pH range between 6.1 and 7.0. For pH determination on paper zones spotted with natural indicator, see Figure 2.

The reliability of the colorimetric response recorded with the natural indicator was compared to the pH values measured using a universal pH strip. Basically, twelve standard solutions were prepared in a pH range between 1 and 12 and sequentially measured using both universal pH strip and paper zones spotted with natural indicator. As it can be seen in Figure 3a, the data recorded with the proposed device are in good agreement with the values achieved using the universal pH strip. Two important features are related to the sample volume required for reading and the pH resolution. The required volume to fill the entire zone in the paper-based devices is 5 µL, contributing to the minimal generation of waste over multiple assays. 

**Figure 4:**
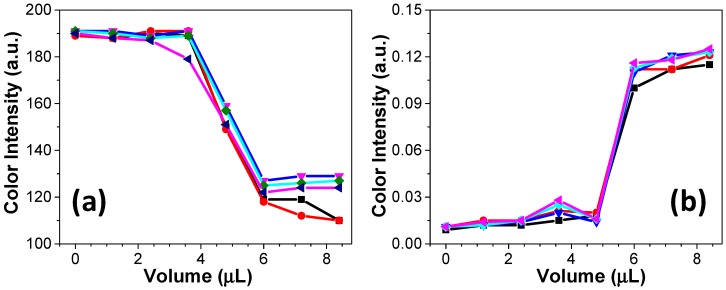
Titration curves performed on wax printed paper microzones showing examples of (**a**) NaOH versus HCl and (**b**) HCl versus NaOH titrations. In both examples, titrations were carried out using equimolar solutions (0.1 mol/L). For each titration, microzones were first spotted with 5 µL of the natural indicator. In (a,b), the color intensities were recorded after adding 1.2 µL aliquots of HCl and NaOH, respectively. Color intensity was captured by smartphone and analyzed through the Photometrix^®^ App.

Once the good agreement between the pH values measured by universal pH strips and paper zones spotted with natural indicator was demonstrated, the pH resolution was also investigated. Basically, different buffer solutions composed of sodium phosphate were prepared in the pH range between 6.1 and 7.0 with increments of 0.1. The recorded data are depicted in Figure 3b. As it can be noted, the color intensity ranged from ca. 0.35 to ca. 0.18 arbitrary units (a.u.) when the pH was raised from 6.1 to 7.0. The data presented in Figure 4 reveal good ability to differentiate pH values in increments of 0.1. This parameter can be defined as the pH resolution of the proposed approach. In comparison with universal pH strips, the resolution achieved with paper microzones is quite interesting and advantageous. It is well-known that universal pH strips are limited to distinguishing pH variations of 1 unit. In this case, the higher resolution of the proposed device opens the possibility to monitor reactions that promote changes in a narrower pH range. 

### 3.3. Acid–Base Titrations on Paper

After calibrating the colorimetric response, two examples of acid–base titrations were tested. The titrations of an NaOH solution versus HCl solution as well as the titration of an HCl solution versus NaOH solution were performed using equimolar concentrations (0.1 mol/L each). According to the data presented in Figure 4a,b, the addition of acid or base aliquots promoted noticeable changes in the colorimetric response. The obtained curves present similar profiles to those obtained using standard volumetric methods (data not shown). The colorimetric responses for both examples demonstrated in Figure 4 were recorded in different color channels. For the titration of base versus acid, the channel S was selected. On the other hand, the titration of acid versus base was monitored in channel H. This channel provides information about the color hue in the ROI, allowing, for example, to distinguish red from yellow [24]. It is important to note that the titration on paper microzone does not require more than 20 µL. Considering the volume usually consumed in a standard volumetric titration (10–50 mL), the waste generation is extremely minimized, thus positively contributing to the green chemistry. In addition to the reduced consumption of reagents, the required time to complete a titration on paper microzones as well as the requested instrumentation are other advantageous features in comparison with standard titration. The proposed procedure is performed within 5 min, and it requires only a micropipette and a smartphone. 

For both examples displayed in Figure 4, five successive titrations were performed in five different microzones. The achieved curves in each titration were overlapped to show a good reproducibility offered by the proposed method. In the titrations of acid versus base and base versus acid on paper, the colorimetric analysis through Photometrix^®^ revealed that the required volume to reach the endpoint of the titration was ca. 5 µL, as expected. In comparison with a previous report from Karita and Kaneta [25], the titration on paper microzones requires a longer time to be completed, and it demands a lower titrant volume. Additionally, the proposed method allows the determination of the titration endpoint and the monitoring of the color intensity in a wide pH range.

### 3.4. Determination of Acetic Acid in Vinegar Samples

The feasibility of acid–base titrations on paper and colorimetric monitoring via smartphone was investigated in the quantitative analysis of acetic acid in three commercial vinegar samples. This application was selected due to the simplicity of the matrix and experiment to demonstrate principles of quantitative analysis for students of different levels or courses. This example is usual in most of the experimental practices of general and analytical chemistry courses. The acid acetic concentration levels were calculated based on the required volume to reach the endpoint of the titration, which was found through the first derivative of the titration curve (data not shown). The achieved results were compared to the values determined by conventional acid–base titration. The obtained concentrations through both methods are shown in Table 1. 

According to the presented data, the differences between values of the proposed method and classical method were lower than 8%, which is considered satisfactory for analytical purposes. The data found by both methodologies were statistically compared through the Student’s *t*-test [31]. Considering that the calculated values for *t*-test (2.58–2.94) were below the theoretical critical value (*t*_crit_ = 3.18), it can be inferred that both methodologies did not differ statistically from one another at a confidence level of 95%. 

It is important to note that the quantitative analysis performed on wax printed microzones was based on the required volume to reach the endpoint of the titration. On the other hand, in the report proposed by Karita and Kaneta [25], µPADs were designed to detect specific samples in different concentration levels. In their study, the authors developed µPADs for detecting NaOH in two concentration ranges (0.1–1.0 mol/L and 0.01–0.10 mol/L), which required different amounts of indicator to make the detection of the titration endpoint possible. In this way, the use of a wax printed zone for monitoring acid–base titrations is simpler than the µPAD proposed by Karita and Kaneta [25]. As previously mentioned, titration can be performed in a single zone, and it does not require either change of indicator or its concentration. 

## 4. Conclusions

In summary, the monitoring of acid–base titrations on paper platforms with a smartphone equipped with a free App showed instrumental and operational simplicity, low cost, and extremely attractive benefits for the environment. Analytical information about the color intensity can be achieved in real-time, thus reducing the analysis time associated with the image processing and pixel’s intensity on similar platforms using scanner as detector [32]. The required volume of samples and reagents was ca. 1000-fold lower than the amount usually employed in standard volumetric methods. Consequently, it is possible to conclude that the waste generation is minimal. In addition, the use of a natural pH indicator over a wide pH range allowed to report standard volumetric applications usually used in chemistry laboratories with acceptable performance. It is important to highlight that the proposed methodology demonstrated the capacity to perform quantitative analysis with high reliability and without statistical difference from a conventional methodology applied worldwide. The mentioned instrumental and economic advantages open new gates that can directly impact analytical chemistry knowledge diffusion at all levels, not requiring sophisticated infrastructure for volumetric analysis.

## Figures and Tables

**Table 1:** Comparison of the acetic acid levels achieved in vinegar samples through classical acid–base titration and the proposed method using wax printed paper-based microzones (*n* = 3).

Samples	Standard Method	Paper Zones
#A	4.25% ± 0.07% (*v*:*v*)	3.93% ± 0.19% (*v*:*v*)
#B	4.14% ± 0.02% (*v*:*v*)	3.87% ± 0.18% (*v*:*v*)
#C	4.11% ± 0.02% (*v*:*v*)	3.92% ± 0.11% (*v*:*v*)

## Supplementary Materials

The following are available online at www.mdpi.com/2072-666X/8/5/139/s1, Table S1: Chemical composition of the solutions prepared* at pH range between 1 and 12.
