# Supplementary material for: Monitoring Acid–Base Titrations on Wax Printed Paper Microzones Using a Smartphone
Source: Micromachines (Basel). 2017 May 2;8(5):139. doi: 10.3390/mi8050139 (PMC6190109; doi:10.3390/mi8050139)
Supplement: Supplementary file 1 [file micromachines-08-00139-s001.pdf]

Supplementary Materials: Monitoring Acid–Base Titrations on Wax Printed Paper Microzones Using a Smartphone

Sandro A. Nogueira, Lucas R. Sousa, Nathália K. L. Silva, Pedro H. F. Rodrigues and Wendell K. T. Coltro

The calibration of the colorimetric response over a pH range was performed using standard solutions prepared according to the description listed in Table S1.

Table S1. Chemical composition of the solutions prepared* at pH range between 1 and 12.

pH	Solution A			Solution B		
	**Composition**	**Concentration (mol/L)**	**Volume (mL)**	**Composition**	**Concentration (mol/L)**	**Volume (mL)**
1	KCl	0.2	25	HCl	0.2	67
2	KCl	0.2	25	HCl	0.2	6,5
3	$KC_8H_5O_4$	0.1	50	HCl	0.1	22.3
4	$KC_8H_5O_4$	0.1	50	HCl	0.1	0.1
5	$KC_8H_5O_4$	0.1	50	NaOH	0.1	22.6
6	KH_2PO_4	0.1	50	NaOH	0.1	5.6
7	KH_2PO_4	0.1	50	NaOH	0.1	29.1
8	KH_2PO_4	0.1	50	NaOH	0.1	46.1
9	$Na_2B_4O_7$	0.025	50	HCl	0.1	4.6
10	$Na_2B_4O_7$	0.025	50	NaOH	0.1	18.3
11	$NaHCO_3$	0.05	50	NaOH	0.1	22.7
12	K_2HPO_4	0.05	50	NaOH	0.1	26.9

*The final solution was prepared by mixing of the indicated volumes for reaching the desirable pH value. Final volume = 100 mL.
